# ACGME Clinical and Educational Work Hour Standards: Perspectives and Recommendations from Emergency Medicine Educators

**DOI:** 10.5811/westjem.2017.11.35265

**Published:** 2017-12-22

**Authors:** Stephen J. Wolf, Saadia Akhtar, Eric Gross, David Barnes, Michael Epter, Jonathan Fisher, Maria Moreira, Michael Smith, Hans House

**Affiliations:** * University of Virginia School of Medicine, Department of Emergency Medicine, Charlottesville, Virginia; † Mount Sinai Beth Israel, Icahn School of Medicine at Mount Sinai, Department of Emergency Medicine, New York, New York; ‡ University of California Davis School of Medicine, Department of Emergency Medicine, Sacramento, California; § Maricopa Medical Center, Department of Emergency Medicine, Phoenix, Arizona; ¶ University of Arizona College of Medicine- Phoenix, Maricopa Medical Center, Department of Emergency Medicine, Phoenix, Arizona; || Denver Health Medical Center, Department of Emergency Medicine, Denver, Colorado; # University of Queensland/Ochsner Health System, Department of Emergency Medicine, New Orleans, Louisiana; ** University of Iowa Carver College of Medicine, Department of Emergency Medicine, Iowa City, Iowa; †† Council of Emergency Medicine Residency Directors, Irving, Texas; ‡‡ American College of Emergency Physicians, Irving, Texas

## Abstract

**Introduction:**

The American College of Emergency Physicians (ACEP) and the Council of Emergency Medicine Residency Directors (CORD) were invited to contribute to the 2016 Accreditation Council for Graduate Medical Education’s (ACGME) *Second Resident Duty Hours in the Learning and Working Environment Congress*. We describe the joint process used by ACEP and CORD to capture the opinions of emergency medicine (EM) educators on the ACGME clinical and educational work hour standards, formulate recommendations, and inform subsequent congressional testimony.

**Methods:**

In 2016 our joint working group of experts in EM medical education conducted a consensus-based, mixed-methods process using survey data from medical education stakeholders in EM and expert iterative discussions to create organizational position statements and recommendations for revisions of work hour standards. A 19-item survey was administered to a convenience sample of 199 EM residency training programs using a national EM educational listserv.

**Results:**

A total of 157 educational leaders responded to the survey; 92 of 157 could be linked to specific programs, yielding a targeted response rate of 46.2% (92/199) of programs. Respondents commented on the impact of clinical and educational work-hour standards on patient safety, programmatic and personnel costs, resident caseload, and educational experience. Using survey results, comments, and iterative discussions, organizational recommendations were crafted and submitted to the ACGME.

**Conclusion:**

EM educators believe that ACGME clinical and educational work hour standards negatively impact the learning environment and are not optimal for promoting patient safety or the development of resident professional citizenship.

## INTRODUCTION

The Accreditation Council for Graduate Medical Education (ACGME) aims to assure a safe learning environment for residents, in part by trying to mitigate fatigue-related medical errors and promote learner wellness.^1^,^2^ To this end, in 2003 the ACGME broadly enacted duty hour standards as part of their common program requirements. However, in 2008, with ongoing patient safety concerns, the Institute of Medicine (IOM) published “Resident Duty Hours: Enhancing Sleep, Supervision, and Safety.”^3^ This highly publicized report called for more stringent resident work-load and duty hour limitations to better protect patients. Shortly thereafter, the ACGME published their 2011 Duty Hour Standards: Enhancing Quality of Care, Supervision and Resident Professional Development and revised their work hour standards within their core program requirements (Table 1).^4^,^5^

Even before these revisions, studies evaluating the benefit of work hour limitations demonstrated mixed outcomes.^6^,^7^,^8^,^9^,^10^ One study on patient safety found longer resident work hours to be associated with increased patient length of stay and the number of intensive care unit transfers, but found no association with inpatient mortality or 30-day readmission rate.^11^ Others suggested scheduling adjustments made by some specialties to comply with work hour standards resulted in increased physician handoffs,^12^,^13^ creating barriers to efficient patient care.^14^,^15^ Residency training programs report significant challenges trying to balance work hour restrictions and enforcement with patient care and educational experiences.^14^,^16^,^17^ Even residents themselves have questioned the benefit of work hour restrictions, as one recent study suggests that limitations do not change resident burnout or self-reported fatigue.^18^

In an attempt to improve resident education, the ACGME held its second *Resident Duty Hours in the Learning and Working Environment Congress* in March 2016. The ACGME invited 64 national organizations to submit position papers with recommendations to improve the work hour standards and the learning environment, from which 56 were invited to provide oral testimony to the Congress. The American College of Emergency Physicians (ACEP) and the Council of Emergency Medicine Residency Directors (CORD) were invited to testify on behalf of emergency medicine (EM). In 2017 the ACGME used this input to revise their common program requirements in an effort to improve the learning and working environment for residents.^5^

With this article, we describe the processes and outcomes by which ACEP and CORD collaborated and jointly explored the state of opinion of EM educators on the ACGME’s clinical and educational work hour standards, and developed recommendations for the 2016 ACGME *Resident Duty Hours in the Learning and Working Environment Congress*.

**Population Health Research Capsule**

What do we already know about this issue?

ACGME duty hour standards have been in place since 2003. Their effects on the EM learning environment have not been extensively reported.

What was the research question?

We sought to gain the perspectives of EM educators and make recommendation regarding the future of ACGME work hour standards.

What was the major finding of the study?

Aside from promoting resident wellness, EM educators largely perceive current work hour standards to have a negative impact on patient safety and the educational experience.

How does this improve population health?

Future revisions of ACGME clinical and work hour standards should aim to prioritize all aspects of the learning environment.

## METHODS

In response to the ACGME request for organizational position papers and recommendations on resident learning and the working environment, ACEP and CORD engaged in parallel but collaborative efforts to generate informed, consensus-based responses from both organizations. The University of Virginia Institutional Review Board reviewed the completed project retroactively and deemed this descriptive report to be exempt from review.

In 2016 an 11-member working group of experts in graduate medical education (GME) and EM residency training convened and engaged in iterative discussions to offer EM’s recommendations for future changes to the dimensions of resident work hour requirements and standards governing key aspects of the learning and working environment. Work group members were purposefully selected through an unstructured discussion process by ACEP academic affairs committee leaders (i.e., authors SW, EG, and HH) for their understanding of, and expertise in, GME and to assure diverse opinions on the learning environment from programmatic, institutional, and national perspectives.

Following broad discussion and commentary, the work group developed and administered a 19-item survey to gain feedback regarding the impact of existing ACGME duty hour standards on EM programs in the areas of patient care and safety (five items), programmatic and personnel costs (six items), consultant and EM resident caseload (four items), and educational experience (four items). Respondents were asked to rate the impact of the 2011 ACGME duty hour standards on a bi-directional 5-point Likert scale from significant negative impact to significant positive impact. Comments were solicited within each area of impact (Appendix).

The survey was distributed to a convenience sample of EM GME stakeholders via the CORD organizational email listserv for EM residency programs. This listserv has participation from each of the 167 allopathic and 32 osteopathic accredited EM training programs. These 199 EM GME programs average five listserv members per program for a total listserv membership of 1,034. Members include departmental chairs, vice-chairs, program directors, associate program directors, educational faculty, and program coordinators. We performed survey data analysis using simple descriptive statistics. Comparative statistics were used to highlight significant differences as appropriate. We identified representative comments in each of the four impact areas to exemplify perceived impact in each of the areas.

Informed by relevant resources,^4^ the work group’s experience, and survey and comment data, the work group engaged in unstructured iterative discussion to develop draft position statements and recommendations in three areas requested by the ACGME: a formal position on current ACGME resident duty hour requirements; dimensions of duty hour requirements; and standards governing key aspects of the learning and working environment. The draft statements were combined, edited and refined to generate independent, consensus-based final recommendations from ACEP and CORD. Each organization’s respective board of directors approved their final recommendations prior to submission to the ACGME.

## RESULTS

The work group was comprised of four women, four active program directors and six past program directors. All members contributed substantially to the iterative discussions. One hundred fifty-seven EM educational leaders responded to the survey representing 15.2% (157/1,034) of the broader listserv membership. Ninety-two of the 157 (92/157, 58.5%) respondents were program directors, yielding a targeted response rate for EM program directors of 46.2% (92/199). Demographic data, professional positions, and geographic locations of respondents are listed in Table 2. Survey responses are grouped by areas of impact and are presented in Table 3.

The impact of the ACGME duty hour standards is reported to have had a majority negative effect in all four domains (i.e., patient care and safety, programmatic costs and personnel, resident case load and competency, and educational experience), although the magnitude of negative impact was least in most of the education experience categories. The only positive impact found was fostering resident work-life balance and wellness. Representative comments selected from 233 completed text fields pertaining to individual areas of impact are presented in Table 4. The final ACEP and CORD formal position statements on ACGME duty hour standards and recommendations for future changes in both the dimensions of duty hour regulation and standards governing key aspects of the learning and working environment are listed in Table 5.

## DISCUSSION

Informed by feedback from EM GME educators, our collaborative, consensus-based process found that the ACGME clinical and educational work hour standards are believed to have overall negative effect on the balance of patient safety and the educational experience. EM educators believe broad work hour regulations have adversely impacted the number of patient handoffs, length of stay, boarding, resident case load, hospital costs, and faculty work load in GME. In addition, the ability of training programs to deliver an effective didactic curriculum and assure resident professional citizenship and accountability has been hampered. The only areas of perceived positive impact were resident wellness and a program’s ability to foster it.

Residency training programs commit to promoting a supportive educational learning environment. In doing so, programs must balance the resident training experience and educational opportunities with resident wellness and patient safety to create a meaningful and effective educational experience. The ACGME strives to ensure this optimal balance through established work- hour standards for residents.^4^ However, EM educators feel that these work hour standards jeopardize the development of personal responsibility and professional accountability that programs work diligently to entrust to their trainees. Furthermore, they are believed to be onerous and cause unnecessary hardship for programs as they monitor and enforce the mandate. *EM educators believe that ACGME work hour standards have historically fallen short of their intended outcomes for patient safety and the educational experience, compromising residency programs’ ability to maintain an ideal learning environment.*

Patient safety is known to be adversely affected by fatigued decision-making, excessive transitions of care, and, in the emergency department (ED), prolonged length of stay and departmental boarding.^3^,^4^,^19^,^20^,^21^,^22^ While ACGME work hour standards are intended to mitigate fatigued decision-making, evidence suggests that they may not be reducing medical errors as expected.^1^,^10^ EM educators believe that work hour standards jeopardize patient safety by increasing transitions across the continuum of patient care and increasing lengths of stay and boarding in the ED. The episodic nature inherent to our specialty’s care allows for EM shift-based schedules to align well with the current ACGME standards. On the other hand, inpatient services do not have the same workflow and frequently are not engaged in straightforward episodic care. For them, the implementation of work hour standards has resulted in an unintended increase in transitions of care and a concomitant loss of patient continuity. EM educators perceive these changes as negatively impacting patient flow in the ED by requiring more handoffs both during the consultation process and in the inpatient setting, creating barriers to efficient and safe patient care across the continuum of care. Additionally, this is believed to have a trickle-down effect of increasing the consultation times, prolonging length of stay, and increasing ED boarding.^23^ Given that previous studies have linked ED length of stay and boarding of inpatients to increased patient morbidity and mortality,^19^,^20^,^21^,^22^ *EM educators encourage the ACGME to consider specialty specific work hour standards allowing for greater alignment of work hour regulations with individual specialty workflow.*

Resident and faculty attendance at didactic conferences is critical and necessitated by ACGME program requirements.^24^ Unfortunately, overlaying conference attendance requirements on the shift-based paradigm that is typically required to meet clinical and educational work hour requirements dramatically decreases a program’s and resident’s ability to be flexible with educational or clinical time. By functionally locking a resident into very distinct work and didactic obligations with strict work hour parameters, residents are not able to autonomously flex their time to promote personal or career development priorities nor to address their personal learning needs. Residents have limited ability to move clinical shifts without violating work hours or compromising conference attendance. Ideally, any standards would afford programs and residents a degree of flexibility to allow individual educational experiences to be maximized.

Both schedule alterations necessary to comply with work hour standards and monitoring of clinical and educational hours have had an additional economic impact on institutions. First, the cost of replacing off-service trainees who are repatriated to their home training programs to fulfill service obligations can be substantial. There are no specific data to determine the amount lost; however, surrogate costs are available. For example, providing just eight hours of care daily by advanced practice providers in the ED can result in substantial costs to a department or organization. It stands to reason that similar effects are felt by other specialties as their resident workforce hours are decreased. Institutions potentially need to re-allocate dollars to fund coverage for changes created by the duty hour standards, shifting funding away from educational programs. Thus, a system has been created by which there is less funding for education without a definitive increase in patient safety or training effectiveness.

Given the increased administrative burden of logging and monitoring resident time, many residency programs have needed to expand their administrative support.^17^ Hidden costs for both residency programs and GME offices in order to meet this unfunded mandate cannot be ignored. Compliance has required in some cases that programs purchase electronic management systems and devote faculty and administrator time to review and monitor data. Some have argued that savings from work hours-related improvements in patient safety may justify the increased personnel and administrative costs.^25^ However, *EM educators still believe the ACGME must explore ways to decrease the programmatic administrative burden of monitoring work hour standards compliance.*

Another significant concern is the notion that the current emphasis on work hour monitoring appears to engender a “clock punching” mentality, de-emphasizing service, professional citizenship, and personal investment in one’s craft – all critical components of professional development for physicians. Though current requirements allow for continuous work hour limitation exceptions when caring for sick patients, the need to document explanations for these exceptions imposes additional administrative burdens on residents, often resulting in a punitive effect rather than rewarding desired behavior. *EM educators encourage the ACGME to consider greater flexibility in clinical and educational work hour standards to promote resident wellness while allowing for the greater development of professional citizenship.*

Currently, two large studies investigating the impact of flexible duty hours on resident training are granted work hour waivers.^26^,^27^ Initial data from the Flexibility in Duty Hours Requirements for Surgical Trainees (FIRST) Trial suggest that increased work hour flexibility was not associated with worse patient outcomes or decreased satisfaction with residents’ own well-being or the quality of their education.^26^ Interestingly, while program directors in this trial perceived more positive effects on safety of patient care, continuity of care, and residents’ ability to attend educational activities, they felt flexible work hours had a positive effect on resident well-being.^28^

The second trial, Individualized Comparative Effectiveness of Models Optimizing Patient Safety and Resident Education (iCOMPARE), is also a large, multi-institutional study designed to evaluate the efficacy and safety of less restrictive work hours in internal medicine training programs.^27^ Importantly, this study will evaluate the impact of relaxed work hour restrictions on the measures of patient safety and trainee education. Data from both of these studies will help to inform future clinical and educational work hour restrictions in all specialties.

In spring 2017, the ACGME announced revisions to subsections of the common program requirements pertaining specifically to the regulation of the learning and working environment.^5^ These revisions place greater emphasis on patient safety, quality improvement, supervision and accountability, resident and faculty well-being, and professional development. Simultaneously, they aim to provide greater flexibility to programs and residents in defining their own learning and working environment, minimizing the burdensome documentation requirements for residents and programs alike.^5^ The impact of these revisions is not yet known. Moreover, the changes to the work hour rules do not address all the concerns identified by the EM community as outlined in our work. Consequently, opportunities exist to determine the specific impact of the more restrictive EM, as compared to non-EM, work hour requirements on ED patient safety and the professional development of EM residents.

## LIMITATIONS

Our informed consensus-based process for developing recommendations for the ACGME was limited in several ways. First, our survey instrument was primarily derived using input from workgroup members with expertise in EM and medical education, and their personal experience and understanding of the literature. Given the significant time constraint imposed by the ACGME for each organization’s formal position paper, our survey was informed by a limited literature review and we were not able to confirm response process validity by piloting the survey for readability and clarity. It is possible that important topics were misunderstood or excluded from the survey instrument. However, while the overall survey response rate was low (15.2%), the response rate from program directors—those most likely to be familiar with ACGME regulations and their effect on trainees—was better (46.2%). Still, with less than 50% of program directors responding to the survey, there is the potential that our conclusions do not accurately represent all program directors’ opinions, despite including input from the broader GME community.

Additionally, open comments were solicited and reviewed by the work group allowing for all opinions to be considered. Next, with an average of 5.2 listserv members per program, any given institution could have answered the survey more than once. The respondent characteristics suggest that there was broad response, but there is still a possibility that over-representation from one institution may have affected the survey results. Next, we recognize the possibility of bias affecting our results. The survey instrument was created by a group of medical education experts, all of whom work (or have worked) within the ACGME program requirements. While survey categories and questions could have been biased towards outcomes favored by the workgroup based on their collective experience, the creation of the initial survey instrument was guided by existing relevant literature. Moreover, there was diverse input from several EM stakeholders and qualitative responses were reviewed and incorporated into iterative discussion minimizing the risk of any bias from the small work group. Lastly, our qualitative commentary data was not formally coded, but rather iteratively discussed by the expert working group to inform commentary and to derive position statements and recommendations.

## CONCLUSION

Emergency Medicine educators believe that ACGME clinical and educational work hour standards have historically negatively impacted the learning environment and do not optimally promote patient safety or the development of resident professional citizenship. EM educators hope that the 2017 revisions to the ACGME clinical work and education standards prioritize all aspects of patient safety, resident wellness, and the ideal learning environment.

## Supplementary Information



## Figures and Tables

**Table 1:** Summary of 2011 & 2017 ACGME clinical and educational work hour standards. 4,5

Standard	Description
Maximum clinical and educational work (duty) hours	80 hours per week (averaged over 4 weeks), inclusive of all in-hospital call activities and moonlighting. (2011) 80 hours per week (averaged over 4 weeks), inclusive of all in-hospital call, at-home call, and moonlighting activities. (2011 & 2017) EM Specific: 72 hours per week (60 clinical hours, plus 12 hours for educational and non-clinical duties). (2011 & 2017)
Maximum continuous clinical and educational work (duty) period length	16 hour limitation for PGY 1 residents (2011 only) 24 hour limitation for PGY 2 and above (Residents may be allowed to remain on site for up to an additional 4 hours for activities related to patient safety, such as care transition, and/or resident education) (2011 & 2017) EM Specific: 12 hour shift limitation (while working in the emergency department) (2011 & 2017)
Maximum in-hospital on-call frequency	No more than every third night, averaged over 4 weeks. (2011 & 2017)
Minimum time off between scheduled clinical and educational work (duty) periods	10 hours off between all duty periods. (2011) 8 hours off between all clinical work or education periods. (2017) 14 hours free after 24 hours of in-hospital call. (2011 & 2017) EM Specific: At least an equivalent period of continuous time off between shifts as the immediately completed scheduled work period.
Mandatory time off from clinical and educational work (duty)	One day (24 hour period) in seven free from all clinical work and required education activities, averaged over 4 weeks. (2011 & 2017) EM Specific: One day (24 hour period) free from all educational and clinical responsibilities every week (no averaging). (2011 & 2017)
Maximum frequency of in-hospital night float	6 consecutive nights.(2011) No limit (2017)
Moonlighting	Not allowed for PGY1 residents. (2011 & 2017) Counts toward 80 hour per week clinical and educational work limit. (2011 & 2017)
Not included in clinical and educational work (duty) hours standards	Reading, studying, and/or academic preparation away from the hospital.

*EM*, emergency medicine; *PGY*, post-graduate year, *ACGME*, Accreditation Council for Graduate Medical Education.

**Table 2:** 2016 ACEP-CORD survey of emergency medical educators perceptions on the impact of the ACGME clinical and educational work hour standards – respondent demographics.

	N (%)
Respondents (total)	157 (100)
Program directors (PDs)	92 (59)
Associate PDs	33 (21)
Assistant PDs	14 (9)
Chairs	4 (3)
Clerkship director	3 (2)
Vice chair	4 (3)
Chief residents	1 (1)
Other	3 (2)
Program geographic location	
East	52 (34)
Midwest	41 (26)
Southeast	35 (23)
Southwest	7 (5)
West	18 (12)
Program format	
PGY 1–3	115 (74)
PGY 1–4	40 (26)

*PGY*, post-graduate year; *ACEP*, American College of Emergency Physicians; *CORD*, Council of Emergency Medicine Residency Directors; *ACGME*, Accreditation Council for Graduate Medical Education.

**Table 3:** 2016 ACEP-CORD Survey of Emergency Medical Educators Perceptions on the Impact of the ACGME Clinical and Educational Work Hour Standards – Quantitative Responses.

Domain	N	Significant negative impact (1)	Negative impact (2)	Neutral (3)	Positive impact (4)	Significant positive impact (5)	Mean
Patient care/safety impact							
No. of EM-EM handoffs	157	10	44	101	2	0	2.61
No. of consultant-consultant handoffs	156	36	67	49	4	0	2.13
Consultant competency	156	14	56	75	10	0	2.52
ED LOS	157	17	67	70	3	0	2.38
ED boarding	157	31	54	67	5	0	2.29
Programmatic costs/personnel impact							
Departmental clinical operations costs	157	15	59	81	2	0	2.45
Hospital clinical operations costs	154	27	86	38	2	1	2.12
Educational leadership (e.g., FTEs)	156	15	66	70	4	1	2.42
Educational administration (e.g., FTEs)	156	20	68	64	3	1	2.34
Faculty workload	157	23	73	57	4	0	2.27
Resident workload	157	12	53	54	34	4	2.78
Resident case load impact							
No. for cognitive competency – EM residents	156	4	33	118	0	0	2.74
No. for cognitive competency – consultants	153	17	75	60	1	0	2.29
No. for procedural competency – EM residents	156	4	34	118	0	0	2.73
No. for procedural competency – consultants	152	14	81	57	0	0	2.28
Educational experience impact							
Effective delivery of a didactic curriculum	156	9	58	81	6	2	2.58
Foster professional citizenship/accountability	156	29	54	68	5	0	2.31
Foster academic involvement/service	155	10	55	70	18	2	2.66
Foster resident work-life balance/wellness	155	4	12	65	68	6	3.39

*ACEP*, American College of Emergency Physicians; *CORD*, Council of Emergency Medicine Residency Directors; *ACGME*, Accreditation Council for Graduate Medical Education; *EM*, emergency medicine; *ED*, emergency department; *No*, number; *LOS*, length of stay; *FTE*, Full-time equivalent.

**Table 4:** 2016 ACEP-CORD Survey of Emergency Medical Educators Perceptions’ on the Impact of the ACGME Clinical and Educational Work Hour Standards – Representative Comments.

Domain	Comment
Patient care and safety	Decreased [duty] hours have led to decreased experience of longitudinal care and stabilization of patients. It also leads to increased handoffs and a decreased sense of responsibility to drive the patient’s plan of care forward in an expedited fashion. This leads to longer time to decisions, admissions, discharges and overall increases boarding. There are now increased handoffs among consultants leading to increased transition of care times, decreased knowledge about patients, which all has downstream impact on the care provided in the ED. Boarding is a big issue at most facilities. Often times it is because the inpatient services cannot disposition or discharge patients in a more timely fashion. That may be due to night float or call systems of coverage (but not primary management) as a way to avoid duty hour violations, leaving the bulk of the work to the day teams. This backs up the ED by creating boarders, which ultimately impacts care of new patients arriving to the ED, as well as the stress level and education of the residents working clinically in the ED.
Programmatic and personnel costs	It is a total waste of time to be chasing someone around and filling out reports because they stayed an hour later and then came to conference the next day without enough sleep. This will be their life, so why not practice for it. I am not in favor of 24-hour shifts at all as they are counterproductive on every service, but if the ICU block would be better served by having the ability to do 7 nights in a row and then have 2 days off, vs. 6 nights in a row, one off, then 1 more night, from a ‘wellness’ perspective it definitely matters. If you don’t work nights ( I would imagine most 9–5 administrators do not), then these administrators probably don’t get it, but having worked 20 years of nights it is very disruptive. I think total duty hours, protected time for conference, etc. are a good idea. The residents may have a “better” workload, but they are also seeing less in three years than with the previous rules. The negative impact on educational leadership is more time spent on dealing with duty hours issues and less time spent on the administration of the education components and innovation. Resident workload has decreased and exposure to patients has decreased while faculty workload has increased, thereby decreasing faculty availability for educational opportunities and faculty fatigue. The clinical operations cost has also increased as hospitals have worked to increase APPs’ availability and increase faculty numbers to address holes in schedules.
Resident case load	I think people are still competent, but I think it takes longer to get to that point. Particularly for consultants. Also teaching residents that it is more important to leave on time than to complete care and also negatively impacting sense of ownership. My residents now have a more difficult time transitioning to junior faculty roles as a result of being coddled by the rules. I think things are worse but “sufficient” The number of patients per resident decreased significantly. Our overall effect is that there is no change, but that is because we went from a 3-year to a 4-year program.
Educational experience	Ironically, the requirements for documentation of hours and other ACGME requirements have taken the place of clinical work. The residents should have the power to have more flexibility in their duty hours and scheduling. Safe patient care is enhanced by rested, healthy resident physicians. However, the time and activity each individual needs to stay well is variable and personable. I recognize that some programs at some sites are malignant and would use the flexibility to hurt residents to provide service. However, the vast majority are not and taking the handcuffs off of the creativity with the schedule would likely lead to healthier physicians and better patient care. Consider providing more leeway for “violations” for each resident. At a minimum give a defined number of times they can “violate” so if they want to work a couple extra days in a row so they can have an extended weekend away with family, etc., they can do that. The documentation and reporting requirements have spawned unbelievable amounts of work for programs and for GME personnel and hospital leadership. Great example of “well intentioned” (I guess) regulations being implemented without sufficient examination of the unintended consequences and questionable rationale. I would say, however, that the effect on non-EM rotations has been healthy -- no more 36-hour calls, no residents who were too tired to think or care. On the other hand, residents got a heavy dose of autonomy and responsibility in the old days that they will not get under the current over-supervised regime. The duty hours have also produced a lot of disdain for honest and accurate reporting. While I believe that duty hours have become too cumbersome, inflexible and irrelevant, it has given guidelines and quantification of resident time in order to help achieve a balanced life. Because EM was already shift-work, and already had a more humane approach to training than many medical specialties, we did not see much impact from the duty hours restrictions to our trainees from a clinical perspective. It does make it much more complex and artificially restricted with respect to our non-clinical educational and service obligations (and opportunities).

*ACEP*, American College of Emergency Physicians; *CORD*, Council of Emergency Medicine Residency Directors; *ACGME*, Accreditation Council for Graduate Medical Education; *ED*, emergency department.

**Table 5:** Summary statements from ACEP and CORD submitted for the 2016 ACGME Congress on the Resident Learning and Working Environment.

	ACEP statements	CORD statements
Formal positions on 2011 ACGME resident duty hour requirements	ACEP supports resident duty hour requirements to improve patient safety, promote resident wellness, and enhance learning. At present, ACEP has concerns about the impact of resident duty hour requirements on patient safety, quality of training, and costs. ACEP believes resident duty hours should be revised to better support the educational experience. ACEP believes that the ACGME should explore specialty-specific duty hour requirements for all specialties.	CORD supports the concept of resident duty hour requirements to promote a supportive educational environment with resident wellbeing and patient safety. CORD has concerns about the effect of resident duty hour requirements on patient safety, transitions of care, quality of training, and costs. CORD believes resident duty hours should be revised to better support the educational experience for trainees. CORD recommends that the ACGME establish specialty-specific duty hour requirements for all specialties.
Formal recommendations regarding dimensions of resident duty hour requirements.	ACEP supports the use of evidence-based resident duty hour dimensions to the end that they improve patient safety and resident wellness. ACEP recommends that the ACGME revise the current dimensions to take into account the need for programmatic autonomy and flexibility germane to adult learning and professional development. ACEP recommends absolving residency programs of the administrative burden of monitoring external moonlighting. ACEP recommends that the ACGME revise these dimensions in a way that maximally promotes and fosters professional citizenship, patient accountability and academic service.	CORD supports duty hours that will enhance patient safety and resident wellness. CORD recommends the ACGME provide more flexibility in duty hours to provide for resident scheduling flexibility and professional development. CORD recommends absolving residency programs of monitoring external moonlighting hours. CORD recommends revising duty hours to promote professional citizenship, patient accountability and academic service.
Formal recommendations regarding standards governing key aspects of the learning and working environment.	ACEP supports efforts to study the effects of relaxing duty hours monitoring and reporting. ACEP recommends that all trainees not on EM rotations be limited to 24 hour continuous scheduled duty hours, regardless of their level of training. ACEP supports a minimum rest interval between duty hour periods for shifts twelve hours or less, and a 14-hour rest period after shifts exceeding 24 hours. Rotating residents should be subject to the duty hour standards of the host rotation program.	CORD endorses further research to determine the value of a change in the frequency of oversight of monitoring duty hours and their reporting. CORD endorses a maximum shift length for all trainees of 24 hours of continuous duty. This would apply to hospital-based rotations on floors and critical care units but be exclusive of the emergency department where maximum shift length would remain 12 hours. CORD endorses a 14 hour period of time off for a shift length of 24 hours. For those shifts that are 12 hours or less, a minimum period of time off is expected between shifts. CORD endorses that residents rotating from outside the department’s home program should be held to the same duty hour standard(s) that apply to the service they are rotating on.

*ACEP*, American College of Emergency Physicians; *CORD*, Council of Emergency Medicine Residency Directors; *ACGME*, Accreditation Council for Graduate Medical Education.
