# Supplementary material for: ACGME Clinical and Educational Work Hour Standards: Perspectives and Recommendations from Emergency Medicine Educators
Source: West J Emerg Med. 2017 Dec 22;19(1):49–58. doi: 10.5811/westjem.2017.11.35265 (PMC5785201; doi:10.5811/westjem.2017.11.35265)
Supplement: Supplementary file 1 [file wjem-19-49-s001.docx]

**Appendix.** 2016 ACEP-CORD Survey of Emergency Medical Educators Perceptions on the Impact of the ACGME Duty Hours – Survey Questions.

1. Please indicate how you believe the 2011 ACGME duty hours have impacted the following patient care/safety related issues.*
   1. The number of EM-EM patient handoffs.
   2. The number of consultant-consultant handoffs.
   3. The consultant competency to provide requested care.
   4. The patient’s length of stay in the emergency department.
   5. A patient’s likelihood to board in the ED awaiting a hospital bed.
2. Please provide comments.
3. Please indicate how you believe implementing, monitoring and enforcing the 2011 ACGME duty hours have impacted the following programmatic cost and personnel needs.*
   1. Clinical operations cost to your department.
   2. Clinical operations cost to your hospital.
   3. Educational leadership costs (e.g., FTE).
   4. Educational administration costs (e.g., FTE).
   5. Resident workload.
   6. Faculty workload.
4. Please provide comments.
5. Please indicate your sense of the sufficiency of resident case load and opportunities as a result of the 2011 ACGME duty hours.**
   1. EM resident patient encounters in order to be competent in the cognitive domains required for independent practice.
   2. Consultant resident patient encounters to be competent in the cognitive domains required for independent practice.
   3. EM resident patient encounters and procedural opportunities to be competent in the procedural skills required for independent practice.
   4. Consultant resident patient encounters and procedural opportunities to be competent in the procedural skills required for independent practice.
6. Please provide comments.
7. Please indicate how you believe the 2011 ACGME duty hours have impacted your programs ability to:
   1. Effectively deliver a didactic curriculum that meets the ACGME requirements.
   2. Foster EM residents’ professional citizenship and accountability to patients.
   3. Foster EM residents’ involvement in academic and service opportunities.
   4. Foster EM residents’ to maintain a better work-life balance and wellness.
8. Please provide comments.
9. Please indicate if you believe any of the duty hour requirements should be altered/changed.
10. Please indicate if you believe there are duty hour dimensions in the learning and working environment that are NOT monitored that you believe should be monitored.

*Responses solicited using a 5-point Likert scale (Significant Negative Impact (1), Negative Impact (2), No Impact (3), Positive Impact (4), Significant Positive Impact (5)).

**Responses solicited using a 5-point bi-directional scale (Significantly Insufficient (1), Insufficient (2), Right Amount (3), Excessive (4), Significantly Excessive (5)).
